# Cytoskeletal Interactions at the Nuclear Envelope Mediated by Nesprins

**DOI:** 10.1155/2012/736524

**Published:** 2012-02-07

**Authors:** Surayya Taranum, Ilknur Sur, Rolf Müller, Wenshu Lu, R. N. Rashmi, Martina Munck, Sascha Neumann, Iakowos Karakesisoglou, Angelika A. Noegel

**Affiliations:** ^1^Institute of Biochemistry I, Medical Faculty, University of Cologne, Joseph-Stelzmann-Strasse 52, 50931 Cologne, Germany; ^2^Center for Molecular Medicine Cologne, Medical Faculty, University of Cologne, Joseph-Stelzmann-Strasse 52, 50931 Cologne, Germany; ^3^Cologne Excellence Cluster on Cellular Stress Responses in Aging-Associated Diseases (CECAD), University of Cologne, Joseph-Stelzmann-Strasse 52, 50931 Cologne, Germany; ^4^International Graduate School in Genetics and Functional Genomics, University of Cologne, 50931 Cologne, Germany; ^5^Department of Biological Sciences, School of Biological and Biomedical Sciences, University of Durham, Durham DH1 3LE, UK

## Abstract

Nesprin-1 is a giant tail-anchored nuclear envelope protein composed of an N-terminal F-actin binding domain, a long linker region formed by multiple spectrin repeats and a C-terminal transmembrane domain. Based on this structure, it connects the nucleus to the actin cytoskeleton. Earlier reports had shown that Nesprin-1 binds to nuclear envelope proteins emerin and lamin through C-terminal spectrin repeats. These repeats can also self-associate. We focus on the N-terminal Nesprin-1 sequences and show that they interact with Nesprin-3, a further member of the Nesprin family, which connects the nucleus to the intermediate filament network. We show that upon ectopic expression of Nesprin-3 in COS7 cells, which are nearly devoid of Nesprin-3 in vitro, vimentin filaments are recruited to the nucleus and provide evidence for an F-actin interaction of Nesprin-3 in vitro. We propose that Nesprins through interactions amongst themselves and amongst the various Nesprins form a network around the nucleus and connect the nucleus to several cytoskeletal networks of the cell.

## 1. Introduction

The nuclear envelope is a barrier separating the nucleus from the cytoplasm. It consists of two lipid bilayers, the outer nuclear membrane (ONM) which is continuous with the endoplasmic reticulum (ER) and the inner nuclear membrane (INM). Although the ONM is contiguous with the ER, it has several unique integral membrane proteins. The INM is intimately linked with the nuclear lamina, a network of intermediate filament proteins, the lamins, and lamina-associated proteins. The two membranes are separated by a *∼*50 nm wide perinuclear space (PNS) and are connected at the nuclear pore complexes (NPC). In addition to its barrier function the NE provides a link to the cytoskeleton through which the shape of the nucleus and its position in the cell is maintained. Important players in this scenario are the Nesprins [1].

Nesprins (nuclear envelope spectrin repeat proteins) comprise a large family of spectrin repeat (SR) containing type II transmembrane proteins localizing to both nuclear membranes with evolutionarily conserved orthologs in lower organisms including *S. pombe *(Kms1), *D. discoideum *(interaptin)*, C. elegans* (ANC-1, ZYG-12 and UNC-83), and* D. melanogaster* (Msp-300) [2–8]. To date, four proteins belonging to the Nesprin family have been identified in mammals, each encoded by a different gene that gives rise to multiple isoforms. Nesprin-1 and -2 contain an N-terminal actin-binding domain (ABD), a central rod domain with several spectrin repeats and a C-terminal transmembrane KASH (Klarsicht/ANC-1/Syne-1 homologue) domain [9–12]. Nesprin-3 harbors an N-terminal binding site for plectin, a large cytolinker which can interact with intermediate filaments, microtubules and actin filaments, and a C-terminal transmembrane region [13, 14]. Nesprin-4 binds to kinesin-1 and is involved in microtubule-dependent nuclear positioning [15].

Nesprins are also essential components of the LINC complex (linker of nucleoskeleton and cytoskeleton) that traverses the NE to connect the nuclear interior with the cytoskeleton in the cytoplasm. In the LINC complex, Nesprins bind to the C-terminus of the evolutionarily conserved INM transmembrane Sun (Sad1/UNC-84) proteins via the C-terminal polyproline stretch of their KASH domain. The interaction takes place in the PNS, defines its width, and is essential for recruitment of KASH proteins to the ONM [16–18]. Several biologically important functions have been attributed to the LINC complex including nuclear anchorage, nuclear migration, anchoring the MTOC to the nucleus, ciliogenesis, and regulation of chromosome dynamics [19–21]. According to the prevailing “bridging and tethering” model the largest isoforms of Nesprins-1 and -2 in the LINC complexes connect the NE to the cytoskeletal networks by projecting their N-termini 300–500 nm into the cytoplasm, although alternative views begin to emerge [22].

Here we focus on the N-terminal region of Nesprin-1. Nesprin-1 is a *∼*1 MDa protein with 74 predicted spectrin repeats. Several isoforms have been identified, among them Drop1 (aa 1–3032), Nesprin-1-165 (previously Enaptin-1-165, aa 1–1479) and CPG2 (aa 537–1479), the internal isoform GSRP56 (aa 2977–3380), and several C-terminal isoforms, Nesprin-1*α*1 and -1*α*2 and Nesprin-1*β*1 and -1*β*2 [9, 11, 23–27]. Nesprin-1-165 harbors the ABD and the first 11 spectrin repeats, CPG2 encompasses spectrin repeats 3 to 11.

The spectrin repeat is an ancient fold and has already been found in proteins in the amoebozoan lineage [28]. It is a domain composed of three *α*-helices with a number of aromatic residues in the hydrophobic core of the domain typically conserved. Structurally, the spectrin repeat is distinguished from other three-helix domains via its characteristic length, its left-handed twist, and localization of the termini to the distal ends of the domain. Spectrin repeats are best known from the spectrin superfamily of proteins, namely, spectrin, *α*-actinin, dystrophin and utrophin, and more recently the Nesprins, in which they are found together with actin-binding domains of the calponin-homology- (CH-) type, EF-hand-type motifs, and various other domains. Typically, there are several consecutive spectrin repeats in these proteins ranging from 4 in *α*-actinin to 74 in Nesprin-1 which allow to build long, extended molecules and to separate functional domains [27, 28]. They also serve as a platform for cytoskeletal and signal transduction proteins [29]. For example, in Nesprin-1 and -2 distinct C-terminal spectrin repeats interact with lamin and emerin to form a network of interactions at the NE, and Nesprin-2 repeats interact with *α*- and *β*-catenin to influence WNT signaling [26, 30, 31].

Spectrin repeats of the *α*-actinin type can dimerize as in *α*-actinin or in spectrin determining the molecular architecture of the protein whereas for dystrophin and utrophin no dimer formation has been reported [28, 29]. Self-association through spectrin repeats has been shown for a C-terminal Nesprin-1 isoform where two molecules interact with each other through distinct C-terminal spectrin repeats to form an antiparallel dimer [26, 32]. Furthermore, the mechanical properties of spectrin repeats make them ideal candidates as components in structures that are exposed to great mechanical stress, such as the cell cortex, the muscle sarcomere, and stress fibers.

We carried out an analysis of sequences contained in the N-terminal isoform Nesprin-1-165 (previously Enaptin-165 [11]) and investigated possible interactions. We detected interactions between spectrin repeats and an interaction of Nesprin-1 with Nesprin-3. We propose that membrane anchored Nesprin-1 can undergo self-interactions through N- and C-terminal spectrin repeats and may build a Nesprin network or “cytoskeleton” around the nucleus in analogy to the spectrin skeleton that stabilizes the erythrocyte membrane [33]. Nesprin-1 also binds to Nesprin-3 enhancing the network and furthermore connecting the nucleus through this interaction to the intermediate filament network.

## 2. Materials and Methods

### 2.1. Cloning

Fragments of mouse Nesprin-1-165 (Enaptin-165) were PCR amplified with the following primers: Nesprin-1-165-1-286 (5′-GCGAATTCATGGCAACCTCCAGAGCATC-3′ and 5′-GCGTCGACTTCTGTTGAAACTGGGCCAC-3′), Nesprin-1-165-573-858 (5′-GCGAATTCAAATTCATGAGTAAGCACTG-3′ and 5′-GCGTCGACTTAGAGTGTCAAGGATTTCTTAC-3′), Nesprin-1-165-859-1144 (5′-GCGAATTCATAGAGAAGGGCAGCCAAAG-3′ and 5′-GCGTCGACTAGCCATTCAATGGGCTC-3′), Nesprin-1-1145-1431 (5′-GCGAATTCAACCACGACGAGTTAGATATG-3′, and 5′-GCGTCGACTTAGAAGTGGTGAAGCACATAC-3′) and cloned into the EcoRI/SalI site of pGBKT7 (BD Biosciences Clontech, Palo Alto, CA), into pGEX-4T-2 (Amersham, Piscataway, NJ) or into pEGFP-C vectors (BD Biosciences Clontech). A polypeptide encompassing residues 287–572 could not be expressed to detectable levels. Human Nesprin-3 sequences encompassing the first three spectrin repeats which correspond to amino acid residues 1–325 were amplified by PCR and cloned into the BamHI/XhoI site of pGEX-4T-2 to generate GST Ne3 SR1,2,3. We also produced NE3 SR1,2 (aa 1–228), NE3 SR1 (aa 1–103), and NE3 SR2 (aa 104–228) as GST fusion proteins and used thrombin cleavage to release the Nesprin-3 polypeptides. DNA sequences corresponding to residues 1–429 and encoding the first four spectrin repeats were cloned into pGADT7 to generate AD-Nesprin-3-ΔC. For generation of GFP-tagged Nesprin-3-ΔC the fragment was cloned into EGFP-C2, for HA-tagged full-length Nesprin-3 sequences corresponding to aa 1–975 were cloned into KpnI/NotI digested pCMV-HA.

### 2.2. Cell Culture and Transfections

COS7 (monkey kidney fibroblast), A172 (human glioblastoma), HeLa (human epithelial carcinoma), HaCaT (human keratinocyte), and A431 (human epidermal carcinoma) cell lines were grown in high-glucose DMEM (Sigma) supplemented with 10% FBS, 2 mM glutamine, and 1% penicillin/streptomycin. CH310T1/2 cells (embryonic mouse mesenchymal stem cell line) were grown in low-glucose DMEM (Sigma) supplemented with 10% FBS, 2 mM glutamine, pyruvate, and 1% penicillin/streptomycin. Mouse myoblast (C2F3) cells were cultured in low-glucose DMEM supplemented with 20% FBS, 1 mM glutamine, 1% penicillin/streptomycin on collagen-coated plates. All cells were grown at 37°C in a CO_2_ incubator (5% CO_2_). COS7 cells were transiently transfected with Nesprin-1-165-GFP (aa 1–1431) [11], GFP-1-286 (ABD), GFP-573-858, GFP-859-1144, GFP-1145-1431, GFP-Nesprin-3-ΔC (aa 1–437; cloned into the AhdI/SacII site of the EGFP-C2 vector), and HA-Nesprin-3 (aa 1−975; cloned into KpnI/NotI digested pCMV-HA) by electroporation (Gene Pulser Xcell, Bio-Rad). Transfected cells were grown for 10–24 h before further analysis.

### 2.3. Antibodies and Immunofluorescence Microscopy

The following antibodies were used: mouse anti- Nesprin-2 mAb K20-478 directed against the ABD, mouse anti-GFP mAb K3-184-2, affinity-purified rabbit anti-Nesprin-1 ABD, and mAb K-43-322-2 [11, 12, 34]. For generation of mAb K43-322-2 a DNA fragment coding for the C-terminal amino acid residues 1222–1431 corresponding to the last two spectrin repeats of Nesprin-1-165 were PCR amplified using the primers 5′-CGGGATCCAAAATGGAGTTTCTCGAACTGAAGTACCG-3′ and 5′-CCCAAGCTTCATTCTGTCCATCTCGTCTGCTCTTGC-3′ and cloned into the BamHI/HindIII site of pT7-7 for expression [35]. The protein was purified by DEAE column chromatography and used for immunization of mice together with ImmunEasy adjuvant (Qiagen, Hilden, Germany) as described [36].

For immunofluorescence, COS7 cells grown on cover slips were fixed in 3% paraformaldehyde for 15 min at room temperature, followed by permeabilization with 0.5% Triton X-100 for 3 min. After blocking with phosphate buffered gelatine, the cells were incubated with primary antibodies for 1 h at room temperature. The following antibodies were used: monoclonal mouse anti-vimentin (DAKO) and rabbit anti-HA (Roche). The cells were washed three times for five minutes each, the samples were incubated for another 1 h with the appropriate secondary antibodies conjugated to Alexa 488 and Alexa 568 (Invitrogen). Nuclei were stained with 4,6-diamino-2-phenylindole (DAPI, Sigma). The cells were imaged using a confocal laser scanning microscope. The images were processed using TCS-SP5 software (Leica).

### 2.4. Recombinant Protein Purification and Pull Downs

The purification of GST and the fusion proteins and pull down assays were performed as described elsewhere [37]. For pull down assays COS7 cells were transfected with EGFP-C2 alone, EGFP-C2-Nesprin-1-fusions, EGFP-C2-Nesprin-3-ΔC, or HA-Nesprin-3 and harvested 24 h after transfection. Cells were lysed in lysis buffer (50 mM Tris-HCl, pH 7.5, 150 mM NaCl, 1% NP-40, 0.5% sodium desoxycholate, 1 mM DTT, 1 mM benzamidine, and 1 mM PMSF), and the lysate was incubated overnight with GST, GST-1-286, GST-1-165-573-858, GST-165-859-1144, or GST-165-1145-1431 coupled to Sepharose beads (GE Healthcare) as indicated at 4°C on a roller. Beads were washed three times each with PBS and lysis buffer, and the bound proteins were eluted with SDS-sample buffer, resolved with 12% SDS-PAGE, and subjected to western blot analysis.

### 2.5. Western Blot Analysis

Whole-cell lysates were separated on 2–10% or 3–15% gradient SDS polyacrylamide gels, the proteins were blotted onto PVDF membranes using wet blotting methods and processed for antibody probing. The blotting times varied from 48 to 72 hours depending on the size of the proteins to be detected. Equal loading was assessed by Ponceau S staining of the blots. Signals were detected using appropriate horseradish peroxidase coupled secondary antibodies followed by enhanced chemiluminescence.

### 2.6. Actin Cosedimentation Assay

Purified GST-Nesprin-3-SR1,2,3 was eluted in glutathione elution buffer and dialyzed against 1x actin polymerization buffer (100 mM imidazole, pH 7.6, 20 mM CaCl_2_, and 20 mM MgCl_2_). GST-Nesprin-1-165-1-286 was purified and cleaved with thrombin to remove GST to yield ABD NE1. Similarly, Nesprin-3 SR1,2 (NE3 SR1,2), Nesprin-3 SR1 (NE3 SR1), and Nesprin-3-SR2 (NE3 SR2) were released from the GST part by thrombin cleavage and used for the assay. For F-actin cosedimentation assays the proteins were centrifuged at 100,000 ×g at 4°C for 60 min to remove precipitated protein. Equal amounts of proteins were incubated in actin polymerization buffer containing 10 mM ATP and 1 mM PMSF for 30 min at RT with or without actin (5 *μ*M). The assay was performed with pH values between 6.0 and 7.0 as described [38] in order to identify the optimal conditions for the interaction of the Nesprin polypeptides with F-actin. The samples were centrifuged again at 100,000 ×g at 4°C for 60 min. Supernatant and pellet fractions were separated and resuspended in 5x SDS sample buffer. Proteins were resolved by SDS-PAGE (12% or 18% acrylamide as indicated) and stained with Coomassie Brilliant Blue. Actin was isolated from *D. discoideum *[39].

### 2.7. Yeast Two Hybrid Analysis

The methods for performing the yeast two-hybrid assay have been described in detail elsewhere (MATCHMAKER Two-Hybrid System 2 Catalogue no. K1604–1; Clontech). Nesprin-3ΔC sequences were cloned into pGADT-7 encoding the Gal4-transactivation domain (AD), Nesprin-1-165, and its domains were cloned into pGBKT-7 encoding the Gal4-DNA binding domain (BD).

## 3. Results

### 3.1. Detection of N-Terminal Nesprin-1 Isoforms in Various Cell Lines

**Figure 1:**
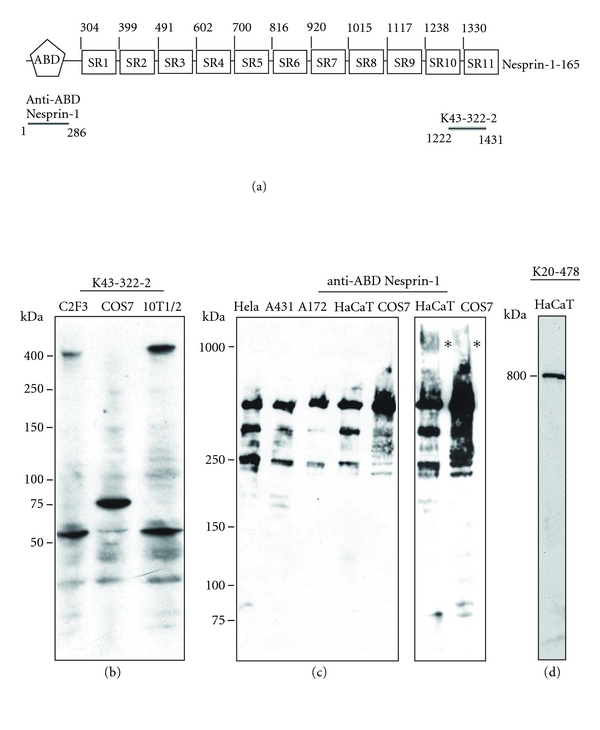
Nesprin-1 isoforms in various cell lines. (a) Schematic of Nesprin-1-165 domain structure and location of the epitopes of the antibodies. ABD, actin binding domain; SR, spectrin repeat. (b) Homogenates of mouse C2F3 myoblasts, COS7, and mouse CH310T1/2 cells were separated on a 3–15% SDS-PA gradient gel and probed after transfer to PVDF membranes with mAb K43-322-2. (c) Homogenates of human cervical carcinoma (HeLa), human epithelial carcinoma (A431), A172, HaCaT, and COS7 cells were separated on 2–10% gradient SDS-PA gels, blotted, and probed for Nesprin-1 Giant using purified rabbit anti-ABD Nesprin-1 antibodies. The separate panel on the right shows a longer exposure for HaCaT and COS7 cell lysates. Stars indicate the position of Nesprin-1 Giant at 1 MDa. (d) HaCaT cell lysate was probed for Nesprin-2 Giant at 800 kDa with mAb K20-478-4 directed against the ABD of Nesprin-2 as a size marker.

Nesprin-1 has several isoforms that perform varied functions in different cell types. The largest isoform Nesprin-1 Giant with a molecular mass of 1 MDa has been reported to be expressed in detectable levels in human fibroblasts and myoblasts [40]. We used monoclonal antibody K43-322-2 that was generated against a recombinant polypeptide harbouring the last two spectrin repeats (aa 1222–1431) of mouse Nesprin-1-165 to study Nesprin-1 isoform expression (Figure 1(a)). This region corresponds to the C-terminus of mouse CPG2 and does not show homology to Nesprin-2 or -3 making the antibody specific for Nesprin-1.

In western blots containing lysates from C2F3, COS7, and CH310T1/2 cell lines mAb K43-322-2 reacted primarily with two proteins. In C2F3 mouse myoblasts a 400 kDa protein and a *∼*50 kDa protein were detected. A similar pattern was obtained for CH310T1/2 cells whereas in COS7 a protein of 75 kDa was seen. Thus the data indicate the presence of N-terminal Nesprin-1 polypeptides of varying size (Figure 1(b)).

For comparison we used polyclonal antibodies directed against the ABD of Nesprin-1 (anti-ABD Nesprin-1, Figure 1(a)) [11]. They recognised in all cell lines tested three prominent bands of *∼*250 kDa and larger molecular weights (Figure 1(c)). We cannot exclude the possibility that the smaller forms are breakdown products of the larger protein. A protein of enormous size (*∼*1000 kDa) which corresponds to Nesprin-1 Giant was detected after longer exposure (Figure 1(c), left, indicated by a star). It was present in all cell lysates and was most prominent in HaCaT and COS7 cells lysates. A HaCaT cell lysate separated on the same gel was probed with mAb K20-478 directed against the ABD of Nesprin-2 to detect Nesprin-2 Giant at 800 kDa as an internal molecular weight marker (Figure 1(d)). The failure to detect the 1 MDa protein with mAb K43-322-2 may be due to a lower affinity of the antibody as compared to the polyclonal antibodies [27].

### 3.2. Interactions of N-Terminal Nesprin-1 Spectrin Repeats

**Figure 2:**
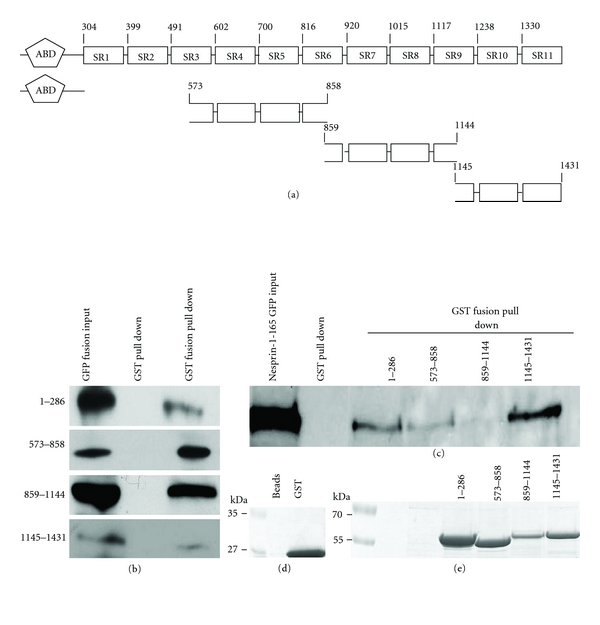
N-terminal spectrin repeats of Nesprin-1 undergo self-associations. (a) Schematic illustration of Nesprin-1-165 constructs used as GST and GFP fusion proteins. Numbers indicate the location of amino acids. (b, c) COS7 cells were transfected with GFP-tagged ABD (aa 1–286), and spectrin repeats of Nesprin-1-165 (aa 573–858, 859–1144, and 1145–1431) (b) and full-length Nesprin-1-165 (c). The cells were lysed in RIPA-buffer and equal amounts of each lysate were incubated with either immobilized GST-fused aa 1–286, 573–858, 859–1144, and 1145–1431 or GST alone for control shown in (d) and (e). The pull down samples were subjected to SDS-PAGE followed by western blot analysis using GFP antibody mAb K3-184. (d, e) The GST-fusion proteins are revealed by Coomassie Blue staining.

Spectrin repeat-containing proteins can form higher-order structures by virtue of interaction among their spectrin repeats; they are also platforms for interaction with other proteins [29]. All Nesprins contain a rod domain with varying number of spectrin repeats, *∼*74 and 56 in Nesprin-1 and -2 Giant, respectively, and eight in Nesprin-3 [14, 27]. Like Nesprin-1, Nesprin-3 also dimerizes as shown by coimmunoprecipitation experiments. Furthermore, it coprecipitates with the ABD of plectin [13]. When we compared Nesprin1's N-terminal spectrin repeats to the ones of mouse *α*-actinin 2 we found that SR1, 2, and 4 had homology with SR2 from *α*-actinin, and SR10 and 11 resembled SR1 and SR4, respectively. The remaining SRs exhibited less homology with the ones of *α*-actinin. To determine whether N-terminal sequences of Nesprin-1 can interact with themselves we used bacterially produced GST-fusion proteins encompassing several spectrin repeats of Nesprin-1-165 (aa 573–858, 859–1144, and 1145–1431; Figure 2(a)) to pull down the corresponding GFP-tagged proteins from COS7 cells. All fusion proteins had the ability to pull down their GFP-tagged counterparts, albeit to differing degrees whereas GST alone did not (Figure 2(b)). We further expressed Nesprin-1-165-GFP in COS7 cells and used the GST-fusion proteins in pull down experiments. In this experiment Nesprin-1-165-GFP was precipitated with GST-573-858 and GST-1145-1431, but not with GST-859-1144 encoding SR7 and 8 and parts of SR6 and 9 which in the comparison showed a lower resemblance to the ones of *α*-actinin. GST alone also did not bind to Nesprin-1-165-GFP (Figures 2(c), 2(d), and 2(e)).

The ABD of Nesprin-1 (GST-1-286) was also included in this assay as similar experiments with the ABD of the Nesprin-3 binding partner Plectin had shown a self-interaction [41]. We found that the ABD of Nesprin-1 indeed could co-precipitate Nesprin-1-165-GFP, and GST-tagged ABD did also pull down GFP-tagged ABD from COS7 cells (Figures 2(b), 2(c), 2(d), and 2(e)). These findings may help to explain the previously described F-actin bundling activity of Nesprin-1's ABD [11]. Likewise, the ABD of Plectin has an F-actin bundling activity [41].

### 3.3. N-Terminal Spectrin Repeats of Nesprin-1 Interact with Nesprin-3

**Figure 3:**
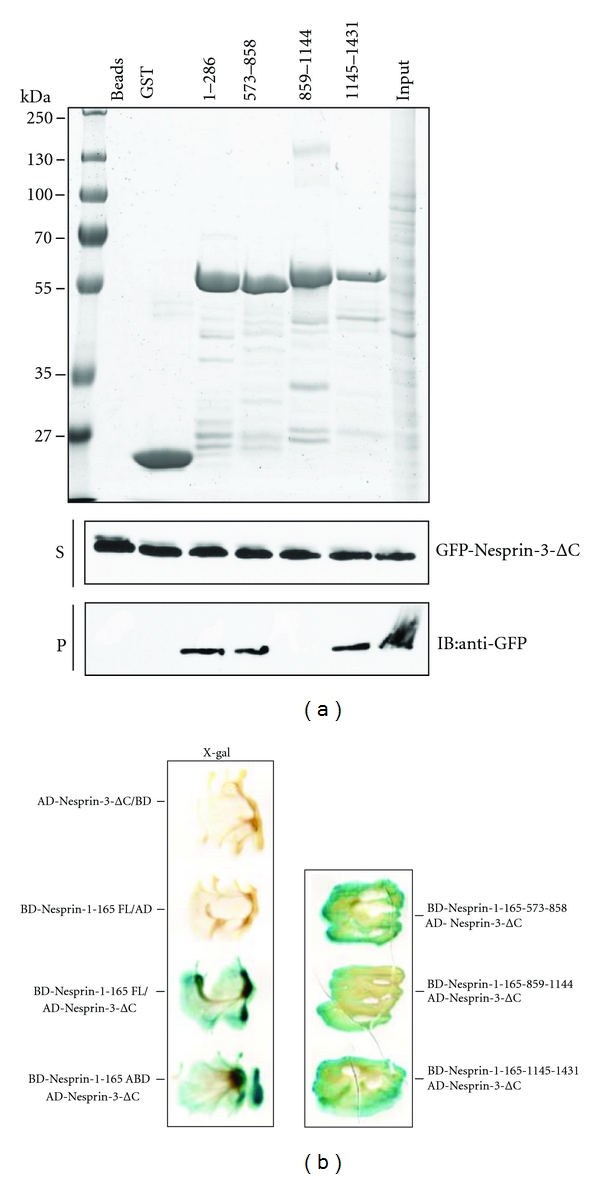
Nesprin-1 N-terminal sequences interact with Nesprin-3. (a) COS7 cell lysates expressing the N-terminal Nesprin-3 (GFP-Nesprin-3-ΔC) fusion protein were incubated with the indicated Nesprin-1-165 polypeptides fused to GST. GST was used as control. The cell lysates (input and supernatant, upper and middle panel) and the pelleted protein (lower panel) were subjected to SDS-PAGE followed by western blotting using mAb K3-184. P, pellet; S, supernatant. (b) Yeast-two hybrid analysis of the interaction between Nesprin-1 and Nesprin-3. Fragments corresponding to Nesprin-1-165 full length and the indicated Nesprin-1-165 polypeptides were fused to the Gal4 DNA binding domain (BD), the N-terminal part of Nesprin-3 lacking the KASH domain (Nesprin-3-ΔC) was fused to the GAL4 activation domain (AD). The corresponding plasmid pairs were cotransformed into yeast strain Y190 and interaction was assessed by filter lift *β*-galactosidase assay. Blue colour development indicates *β*-galactosidase activity.

By pull down experiments we next investigated the possibility of an association between Nesprin-1 and Nesprin-3 through their spectrin repeats. COS7 cells were transfected with a GFP-tagged Nesprin-3 construct (aa 1–437) encompassing SR1–4 but lacking the C-terminal half (GFP-Nesprin-3-ΔC), and the lysates were used for pull downs with the GST-tagged Nesprin-1-165 polypeptides. GST served as negative control for Nesprin-3 binding. We found that all GST-fusion proteins with the exception of GST-859-1144 were able to pull down GFP-Nesprin-3ΔC (Figure 3(a)). Our data suggest that Nesprin-1 associates with Nesprin-3 through several spectrin repeats. Furthermore, the ABD of Nesprin-1 (GST-Nesprin-1-165-1-286) also bound to Nesprin-3.

We performed yeast-two hybrid analysis to obtain additional support for the association between Nesprin-1 and -3. The Nesprin-1 and -3 regions used in the pull down assay were fused with the GAL4 DNA-binding domains of pGBKT7 vector (BD-Nesprin-1-165-ABD, BD-Nesprin-1-165-573-858, BD-Nesprin-1-165-859-1144, BD-Nesprin-1-165-1145-1431) and the GAL4 transcription activation domains in the pGADT7 vector (AD-Nesprin-3-ΔC), respectively. Yeast cells cotransformed with AD-Nesprin-3-ΔC, and the BD-Nesprin-1-165 plasmids could grow on the selection plates and were positive in the *β*-galactosidase test. No interaction was detected in the negative controls (Figure 3(b)). We also had a positive signal with BD-Nesprin-1-165-859-1144, the polypeptide that had not shown interactions with Nesprin-1-165-GFP and GFP-Nesprin-3ΔC in the pull down assays. In general, the yeast-two hybrid assay is a first screen for the identification of novel binding partners which then needs to be confirmed by independent methods. In our case we first carried out two sets of independent experiments and only then used the yeast two-hybrid assay which appears to have given a false-positive reaction.

### 3.4. The Nesprin-1 ABD Binds to F-Actin in the Presence of Nesprin-3

**Figure 4:**
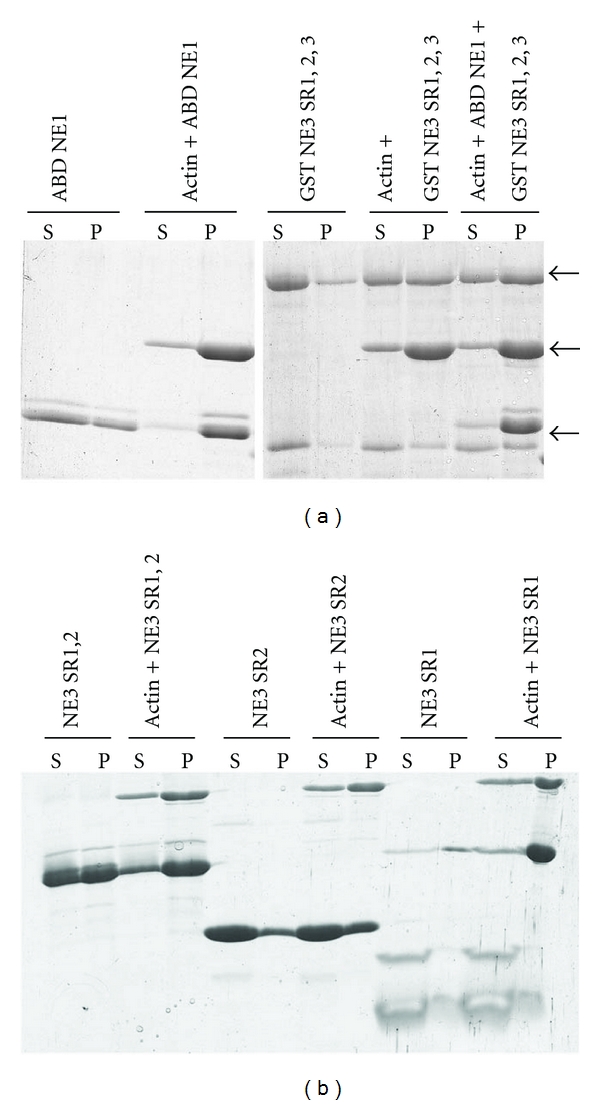
F-actin interaction of Nesprin-1 ABD and Nesprin-3. (a) Nesprin-1 ABD binds to F-actin in vitro in the presence of Nesprin-3 in an F-actin co-sedimentation assay. Recombinantly expressed and purified GST-Nesprin-3 SR1,2,3 (GST NE3 SR1,2,3), and Nesprin-1 ABD (ABD NE1) were precleared and incubated with and without actin at room temperature under polymerizing conditions. High-speed centrifugation at 100,000 ×g for 1 h at 4°C was followed by separation of the protein mixture into supernatant (S) and pellet (P) fractions. ABD NE1 and GST NE3 SR1,2,3 bound to F-actin and were observed in the pellet fraction. F-actin binding was also observed when both proteins were added to the polymerization assay (actin + ABD NE1 + GST NE3 SR1,2,3). Samples were treated with 5x SDS-sample buffer and proteins separated on SDS-PA gels (12% acrylamide) and stained with Coomassie Brilliant Blue. The two closely migrating polypeptides in the Nesprin-1 ABD sample were identified as Nesprin-1 ABD by mass spectrometry. Arrows from top to bottom indicate the location of GST NE3 SR1,2,3, actin, and ABD NE 1. (b) Spectrin repeats of Nesprin-3 bind to F-actin. Nesprin-3 spectrin repeats SR1,2, SR2, and SR1 were used in the assay. Only SR1,2 (26.7 kDa) copelleted with F-actin (40 kDa) whereas SR2 (14.6 kDa) and SR1 (12.1 kDa) stayed in the supernatant. Proteins were separated on SDS-PA gels (18% acrylamide). The fast migrating protein in the SR1 sample corresponds also to SR1.

Based on the findings that the ABD of Nesprin-1 can bind to F-actin with high affinity and can also bundle actin filaments [11] we next asked the question whether the interaction between Nesprin-1 and Nesprin-3 interferes with the F-actin binding activity of Nesprin-1 and performed competitive F-actin co-sedimentation assays in the presence of Nesprin-3 polypeptides. First we identified conditions in which ABD-Nesprin-1 (ABD NE1) would efficiently pellet with F-actin. The ABD of Nesprin-1 has a theoretical pI of 9.0 which suggested to us an ionic interaction between F-actin and the ABD. Therefore we tested the interaction at various pH and found that ABD NE1 co-precipitated efficiently with F-actin at a pH below 7.0. Under these conditions ABD NE1 was nearly completely present in the F-actin pellet after high-speed spin whereas at pH above 7.0 the protein was increasingly present in the supernatant (see Supplementary Figure 1 in Supplementary Material available online at doi:10.1155/2012/736524, and data not shown; Figure 4(a)). We therefore carried out all further experiments at a pH below 7. In control experiments without F-actin the majority of ABD NE1 remained in the 100,000 ×g supernatant. The addition of GST NE3 SR1,2,3 (aa 1–325 fused to GST) did not interfere with the ABD NE1 F-actin interaction. Instead, the protein was also observed in the F-actin pellet (Figure 4(a)).

### 3.5. Nesprin-3 Spectrin Repeats Can Interact with F-actin

We next probed whether GST NE3 SR1,2,3 depends on ABD NE1 in order to coprecipitate with F-actin or whether it directly interacts with F-actin. The latter proposal is not unprecedented. Dystrophin which contains a functional ABD related to the one in *α*-actinin and Nesprin-1 has an additional F-actin interaction site in its spectrin repeats containing rod domain [42, 43]. GST NE3 SR1,2,3 co-precipitated with F-actin in spin down assays whereas in the absence of F-actin the majority of the protein stayed in the supernatant under polymerizing conditions (Figure 4(a)). In order to identify the spectrin repeat(s) of NE3 SR1,2,3 responsible for this activity we used NE3 SR1,2 and SR1 and SR2 which had been released from GST by thrombin cleavage. In these assays SR1,2 did bind to F-actin. In control experiments, under polymerization conditions about 50 percent of NE SR1,2 were present in the 100,000 ×g pellet. Upon addition of actin the majority of the Nesprin-3 peptide pelleted with F-actin. NE3 SR2 did not show an enrichment in the F-actin pellet and NE3 SR1 remained in the supernatant under all conditions (Figure 4(b)). We conclude that NE SR1,2 has the ability to interact with F-actin whereas for SR1 and SR2 we cannot exclude the possibility that a successful F-actin interaction is prevented by inappropriate folding of the polypeptides.

### 3.6. Nesprin-3 Is Able to Recruit Vimentin to the Nucleus

**Figure 5:**
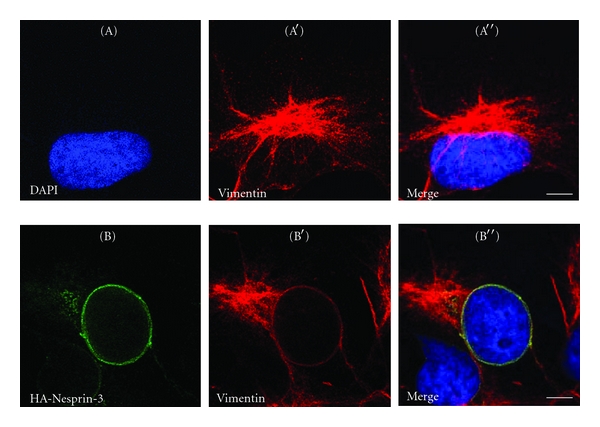
Nesprin-3 recruits intermediate filaments to the nuclear envelope in COS7 cells. COS7 cells stained for vimentin reveal the typical cytoskeletal staining (A-A′′). In HA-Nesprin-3 transfected COS7 cells vimentin was recruited to the NE and co-localized with HA-Nesprin-3 (B-B′′). Confocal images are shown. Size bar, 10 *μ*m.

**Figure 6:**
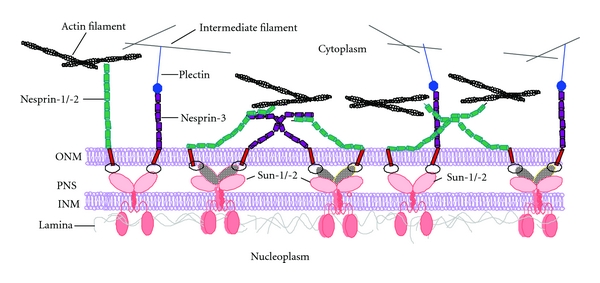
Illustration of Nesprin interactions at the outer nuclear membrane surface. The inner (INM) and outer (ONM) nuclear membranes are shown to allow simultaneous documentation of the interaction of Nesprins with SUN-domain complexes. Nesprins are aligned along the nuclear envelope. Nesprin-1/-2 ABDs interact with F-actin, N-terminal Nesprin-1 spectrin repeats interact with Nesprin-3, the latter can also interact with the ABD of plectin. Therefore, Nesprins integrate the NE with the major cytoskeletal filaments allowing the formation of a multifunctional Nesprin network along the surface of the ONM. INM located Nesprins have been omitted from this scheme. PNS, perinuclear space.

From previous studies it is known that Nesprin-1 and -2 through their association with F-actin can assemble an F-actin cage around the nucleus [44] (our own unpublished observations). We were therefore wondering whether Nesprin-3 in analogy is able to recruit an intermediate filament network to the nucleus. As Nesprin-3 is not strongly expressed in COS7 cells [14], we expressed HA-Nesprin-3 in these cells and probed the localization of vimentin. In untransfected cells we observed a filamentous vimentin staining which was not particularly enriched around the nucleus. However, in cells expressing HA-Nesprin-3 we found vimentin at the nucleus where it colocalized with Nesprin-3 (Figure 5) extending recently reported findings in zebrafish to mammalian cells [45]. Such an arrangement might then allow the formation of a network of several filamentous systems that are connected through interactions between Nesprin-1 and Nesprin-3 (Figure 6). For control we ectopically expressed GFP-fused Nesprin-1 ABD. This protein was recruited to the nuclear envelope but it did not affect vimentin localization (Supplementary Figure 2).

## 4. Discussion

We show here that Nesprin-1 can self-associate through its amino terminal sequences. Earlier studies have demonstrated that the C-terminal isoform Nesprin-1*α* can dimerize by association between its third and fifth spectrin repeats [26, 32]. Further, Nesprin-3*α* was also shown to form dimers. the spectrin repeats involved have, however, not been identified [13]. Our data on the self-interactions of Nesprin-1 N-terminal spectrin repeats lead to the intriguing possibility that an association among Nesprins may not necessarily be confined to the isoforms containing the KASH domain. Other isoforms may behave similarly and thus help target or retain further isoforms at the NE.

The length of the Giant Nesprin isoforms has been calculated to amount to 300 to 500 nm and current models depict them as projecting into the cytoplasm to facilitate nucleocytoplasmic coupling. Our data suggest that self-association and interaction among the N-terminal spectrin repeats of Nesprin-1 and of the much shorter Nesprin-3 (*∼*40 nm) allows their alignment along the NE. Such an arrangement could play a role in the maintenance of the nuclear morphology. Consistent with this hypothesis Nesprin-2 Giant knockout mice show an increase in nuclear size indicating that the protein is important for the NE morphology in primary dermal fibroblasts and keratinocytes [46]. Also, mutations in human Nesprin-1 and -2 adversely affect nuclear morphology [47]. Further, coimmunofluorescence data of Nesprin-2 Giant using antibodies against its N- and C-termini which are far apart reveal a similar location at the nuclear envelope [30]. Thus our data are not consistent with the model showing the Giant Nesprin isoforms as reaching out into the cytoplasm. The nucleocytoplasmic coupling may present an additional function of the ABDs apart from their involvement in nuclear positioning and migration by binding to F-actin. Also, many proteins containing spectrin repeats are known to align along membranes [48].

 We also show here that Nesprin-3 can interact with F-actin in vitro. Similar observations have been made for dystrophin where a region encompassing SR11–15 is responsible for F-actin binding [42, 43]. A sequence comparison showed *∼*19% identity and 37% homology between Nesprin-3 SR1,2,3, and SR13–15 of dystrophin. For other regions these values were lower. Further experiments are needed to show the in vivo relevance of our finding.

Taken together, our data imply the existence of a Nesprin-based meshwork at the NE similar to the oligomeric lattices formed by the SUN-domain proteins at the NE [22, 49]. We suggest that Nesprin-1 oligomerizes through C-terminal and N-terminal spectrin repeats. We further propose that spectrin repeats are also involved in facilitating interactions among Nesprin-1 and -3 and connect the nucleus to the F-actin and intermediate filament networks. We hypothesize that the self-association and interaction among Nesprins favours the formation of a protein network at the NE similar to the spectrin network in the erythrocyte and present the model of a triple layer where the Nesprins as type II transmembrane proteins are anchored in the nuclear envelope through their C-termini. They form a network surrounding the nucleus and link it to the cytoskeletal systems in the cytoplasm (Figure 6). The meshwork could function as a buffer against forces involved in conducting nuclear migration and positioning and thereby making the nucleus less malleable. This is supported by data from peripheral blood leukocytes where the Nesprin-1 and -2 content is strongly reduced [50]. These leukocytes are highly deformable allowing them to squeeze through the vascular endothelial walls. Presumably, the interactions we have described for Nesprin-1 can be extended to Nesprin-2 as it is very similar to Nesprin-1 and is primarily present in tissues and cells which do not express Nesprin-1.
